# Synthesis of Polyaniline Coated Magnesium and Cobalt Oxide Nanoparticles through Eco-Friendly Approach and Their Application as Antifungal Agents

**DOI:** 10.3390/polym13162669

**Published:** 2021-08-10

**Authors:** Suryyia Manzoor, Ghazala Yasmin, Nadeem Raza, Javier Fernandez, Rashida Atiq, Sobia Chohan, Ayesha Iqbal, Shamaila Manzoor, Barizah Malik, Franz Winter, Mudassar Azam

**Affiliations:** 1 Institute of Chemical Sciences, Bahauddin Zakariya University, Multan 60000, Pakistan; suryyia878@gmail.com (S.M.); ghazala31pk@yahoo.com (G.Y.); aishaiqbal561@gmail.com (A.I.); 2 Department of Chemistry, Emerson University, Multan 60000, Pakistan; nadeemr890@gmail.com; 3 Department of Chemical Engineering, University College London, Torrington Place, London WC1E 7JE, UK; j.fernandez.garcia@ucl.ac.uk; 4 Department of Plant Pathology, Bahauddin Zakariya University, Multan 60000, Pakistan; rashidaatiq@bzu.edu.pk (R.A.); sobia_mustafa2006@hotmail.com (S.C.); 5 Department of Physics and Astronomy, University of Florence, Via Sansone1, 50019 Sesto Fiorentino, Italy; 6 School of Biochemistry and Biotechnology, University of the Punjab, Lahore 54590, Pakistan; barizah.ibb@pu.edu.pk; 7 Institute of Chemical, Environmental and Bioscience Engineering, TU WIEN, Getreidemarkt 9, 1060 Vienna, Austria; franz.winter@tuwien.ac.at; 8 Institute of Chemical Engineering & Technology, University of the Punjab, Lahore 54590, Pakistan

**Keywords:** green synthesis, nanoparticles, PANI, *Manilkara zapota* leaves extract, *Aspergillus niger*

## Abstract

Plant-mediated synthesis of nanoparticles exhibits great potential to minimize the generation of chemical waste through the utilization of non-toxic precursors. In this research work, we report the synthesis of magnesium oxide (MgO) and cobalt oxide (Co_3_O_4_) nanoparticles through a green approach using *Manilkara zapota* leaves extract, their surface modification by polyaniline (PANI), and antifungal properties against *Aspergillus niger*. Textural and structural characterization of modified and unmodified metal oxide nanoparticles were evaluated using FT-IR, SEM, and XRD. The optimal conditions for inhibition of *Aspergillus niger* were achieved by varying nanoparticles’ concentration and time exposure. Results demonstrate that PANI/MgO nanoparticles were superior in function relative to PANI/Co_3_O_4_ nanoparticles to control the growth rate of *Aspergillus niger* at optimal conditions (time exposure of 72 h and nanoparticles concentration of 24 mM). A percentage decrease of 73.2% and 65.1% in fungal growth was observed using PANI/MgO and PANI/Co_3_O_4_ nanoparticles, respectively, which was higher than the unmodified metal oxide nanoparticles (67.5% and 63.2%).

## 1. Introduction

*Aspergillus niger* is a filamentous fungus abundantly found in decaying vegetation. A humid environment and a temperature range of 30–35 °C are the most favorable conditions for its rapid growth, which ultimately result in a fast decay of vegetables, causing a great loss to the economy [1]. Moreover, it is also responsible for spoilage of stored food items such as figs, nuts, dates etc. Because of its notorious saprophytic behavior in soil and its widely spread spores in air, *A. niger* is now becoming a significant cause of fungal infection in birds’ respiratory tracts, especially those with low immunity [2,3]. Commonly, synthetic pesticides are a preferable choice to control the fungal attack; however, their non-biodegradability and accumulation in food products make them vulnerable to human health [3]. To further remediate this issue, various other approaches, including adsorption, enzyme catalysis, irradiation, membranes, and miscellaneous chemical methods, have also been reported [4]. Nanotechnology has recently marked its entry in the concerned field, and few of the nanoparticles have been initially explored as antifungal agents [5,6,7]. Due to their considerably large surface area and minor size, nanoparticles exhibit numerous beneficial properties and applications in various methodical fields (textile, agriculture, electronic devices, medicine, and cosmetics) [8,9,10,11,12,13]. Nanomaterials based on a combination of inorganic nanoparticles with polymeric materials specify a new class of substances that show superior performance compared to their inorganic counterparts. These composites enhance biological as well as photodegradable properties [14,15,16,17]. For decades, diverse methods for the synthesis of nanomaterials have been explored, ranging from complex chemical pathways to simple green approaches [18]. Among the prevailing trends, the selection of techniques that are environmentally benign and cost-effective has received prime attention. Hence, the plant-mediated synthesis as a green approach is gaining fame because it omits the use of chemical reagents, and a large number of nanoparticles such as silver, gold, and zinc have been thus far prepared [19,20,21]. The use of plant extracts also results in surface modification of nanoparticles because of various phytochemicals present in them [22].

MgO and Co_3_O_4_ nanoparticles have been synthesized by both chemical and plant-mediated routes and are widely used to evaluate their ability as antibacterial and anticancer agents [23,24,25]. A variety of plants have been utilized for the green synthesis of both nanoparticles. Palanisamy and Pazhanivel successfully applied betel leaves extract to obtain MgO nanoparticles from their respective salts [26]. Similarly, rosemary, Limonia acidissima fruit and Neem leaves extracts have also been applied for the synthesis of MgO nanoparticles [27,28,29]. Similarly, Co_3_O_4_ has been previously prepared using Punica granatum, fenugreek, and citrus leaves extracts [30,31,32].

*Manilkara zapota* leaves extract contains various phytochemicals which can serve as reducing agents as well as particle stabilizers and can possibly be tested for the synthesis of nanoparticles [33]. The major components present in the leaves of *Manilkara zapota* are lupeol acetate, glycosides, flavonoids, oleanolic acid, apigenin-7-O-α-L-rhamnoside, terpenoids, myricetin-3-O-α-L-rhamnoside, and caffeic acid and the extracts have been employed for varied applications [34].

In this work, we have explored, for the first time, the applicability of *Manilkara zapota* leaves extracts for the synthesis of magnesium oxide (MgO) and cobalt oxide (Co_3_O_4_ nanoparticles. These nanoparticles were further modified by coating with polyaniline (PANI). Their polymeric counterparts may prove superior to retard fungal growth, and hence, they were tested to inhibit the propagation of *Aspergillus niger*. PANI-coated magnesium oxide and cobalt oxide nanoparticles have not yet been studied to control the spread of Aspergillus, and hence this is the theme of this work. The main reason for choosing Mg and Co nanoparticles is their substantial role in the phytochemical reactions of certain enzymes in plants. Therefore, the practical implementation of this work is foreseeable, and it can be said that their presence in the environment will not only inhibit the fungal growth but may also prove beneficial in aiding plant growth.

## 2. Materials and Methods

Magnesium nitrate hexahydrate (99%), methanol (99%), cobalt nitrate hexahydrate (98%), KOH (97%), ammonium peroxy disulphate (98%), acetone (99%), and HCl (35.5%) were bought from Merck, Darmstadt, Germany while sodium hydroxide (97%), aniline (99%) and ethanol (99%) were purchased from Riedel-de Haen (Seelze, Germany). Voriconazole (98%) was obtained from Sigma Aldrich (St. Louis, MO, USA).

### 2.1. Green Synthesis of MgO and Co_3_O_4_ Nanoparticles

MgO and Co_3_O_4_ nanoparticles were synthesized using fresh leaves extract of *Manilkara zapota*, commonly known as Chikoo (in local language). Briefly, 20 g of fresh *Manilkara zapota* leaves were collected, washed with deionized water, and cut into little pieces using a sterilized knife. The finely cut leaves were then boiled for 60 min followed by cooling of the extract to ambient condition. The extract was filtered to remove the leaves residues and stored in amber glass bottles kept in the refrigerator for further use.

In the next step, the synthesis of MgO nanoparticles was performed by taking 20 mL of fresh leaves extract and 20 mL of deionized water in a beaker. The solution was heated to 60 °C, and to this, 5 g of magnesium nitrate was added. The temperature was further raised to 80 °C with constant stirring for 4 h. The brown-colored particles were thus obtained, which were washed with deionized water and further heated in the furnace at 500 °C for 1 h, then cooled at room temperature and stored. Similarly, the Co_3_O_4_ nanoparticles were prepared using cobalt nitrate following the same procedure as described above.

### 2.2. Coating of Polyaniline (PANI) on MgO and Co_3_O_4_ Nanoparticles

Surface coating of the nanoparticles (MgO and Co_3_O_4_) with polyaniline was achieved by selecting aniline as a monomer. For this purpose, two types of solutions were prepared. For the preparation of solution A, 0.15 g of MgO nanoparticles were suspended in 35 mL of 0.1 M HCl and were sonicated for 30 min in an ultrasonic bath. Afterward, 1 mL of aniline monomer was added to solution A and was further stirred for 20 min. In the meantime, solution B was prepared by mixing 2.49 g of ammonium peroxodisulfate in 20 mL of 0.1 M HCl. Solution B was added dropwise in solution A with constant stirring for 2 h at 4 °C. The mixture was then kept for 12 h in ambient conditions for polymerization purposes. The coated particles were filtered, washed with deionized water, and dried in a vacuum oven for 24 h at 60 °C.

### 2.3. Characterization

The morphological characterization was performed using a scanning electron microscope (SEM, LEO-1530, Oberkochen, Germany) under high vacuum conditions in a series of magnification ranges. The samples were dispersed in ethanol, and a drop was applied on carbon-coated copper sample holder. The solvent was allowed to evaporate completely before SEM analysis.

FTIR spectra were obtained by Bruker Alpha II spectrophotometer (Billerica, MA, USA), which uses a single reflection diamond ATR module. The scan was performed between 4000–650 cm^−1^. For the purpose of XRD analysis, Bruker D8 (Karlsruhe, Germany) was used. The increment was 0.1° per step with a scan rate of 0.1° per second.

### 2.4. Zone of Inhibition Test

The ability of nanoparticles to act as antifungal agents was investigated against *Aspergillus niger* through the zone inhibition technique. The whole apparatus (peter dishes, pipette tips, and micropipette) was autoclaved. PDA (Potato Dextrose Agar) media was prepared in 500 mL starch obtained from freshly boiled potatoes, to which 25 g of dextrose and 25 g of agar were added. Afterward, 0.5 g of streptomycin was introduced to the PDA media.

Suspensions of PANI/MgO, PANI/Co_3_O_4_, magnesium, and cobalt oxide nanoparticles with four different concentrations (3, 6, 12, and 24 mM) were prepared in an aqueous medium. One-third of the Petri plates was filled by PDA and kept in an incubator for solidification. The suspension of *Aspergillus niger* was prepared and streaked on PDA. A hole was then made in the middle of PDA in each Petri dish with the help of a pipette tip through which different concentrations of nanoparticles were added. A control was also prepared under similar conditions by using a plate with a hole filled only with deionized water in the absence of nanoparticles. These Petri plates were placed in an incubator at 25 °C. The growth of *Aspergillus niger* was accompanied by time intervals of 24, 48 and 72 h.

Furthermore, the antifungal activity of nanoparticles was compared with voriconazole (fungicide) following the same procedure. The zone of inhibition was measured after the same time intervals (24, 48, and 72 h).

## 3. Results and Discussion

**Figure 1:**
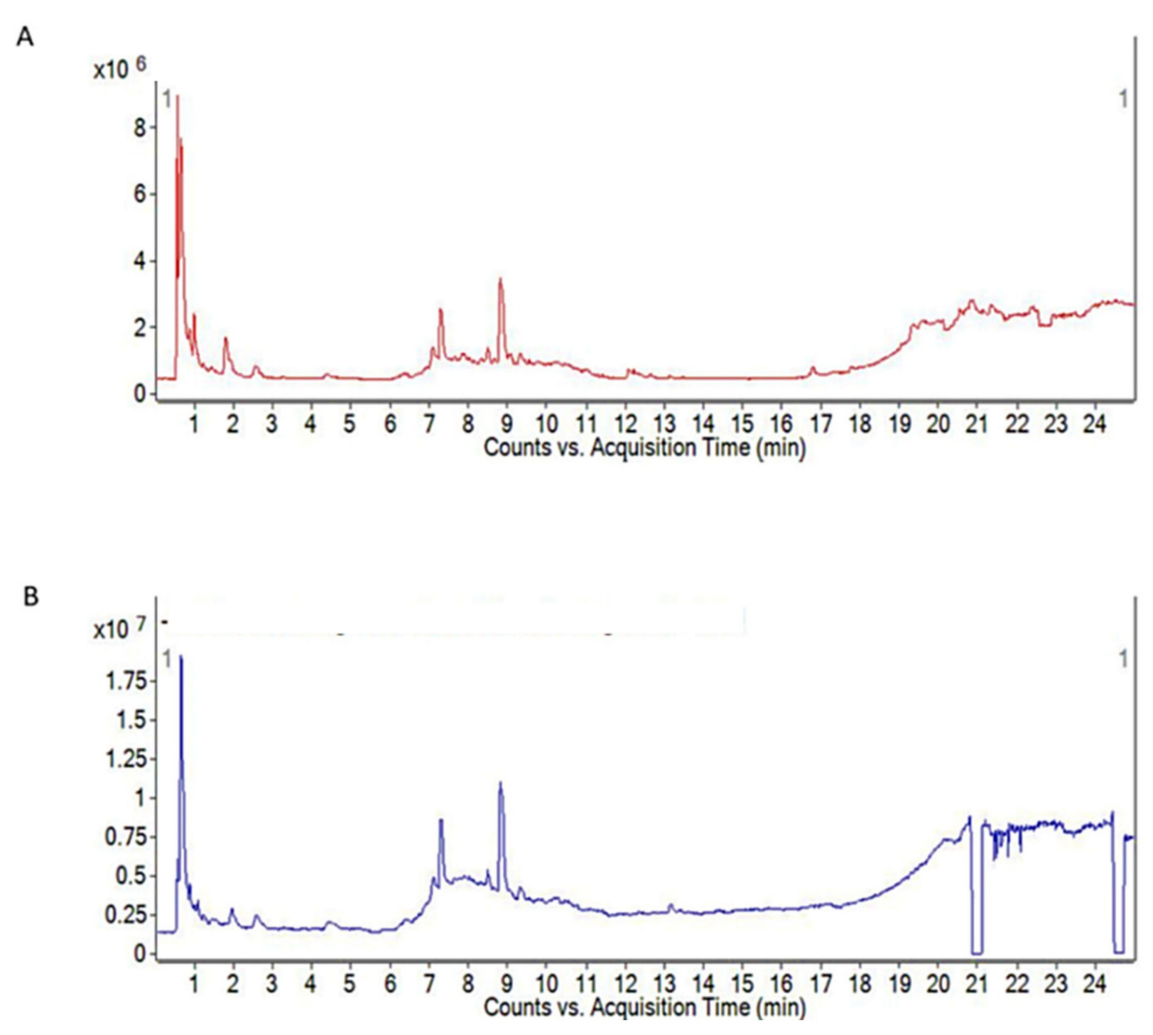
Total ion chromatogram of *Manilkara zapota* aqueous leaves extract by ESI-Q-TOF MS (**A**) positive (**B**) negative ionization modes. Column: Zorbax Eclipse XDB-C18, 2.1 × 150 mm; mobile phase: 0.1% (*v/v*) of formic acid in water and 0.1% (*v/v*) of formic acid in acetonitrile [35].

Plant-mediated synthesis of metal-based nanoparticles is gaining enormous attention due to its environmentally friendly and cost-effective approach. Various phytochemicals present in the extracts of plants are considered to act as capping agents and bio-reductants in converting metal ions to their respective metals [22]. *Manilkara zapota* plant has been widely studied and screened for phytoconstituents present in its different parts. The aqueous leaves extract has been found to possess high levels of phenolics and flavonoids. The literature survey revealed that the aqueous leaves extract has the highest content of these compounds compared to other parts of the plant. Figure 1 shows the total ion chromatogram obtained by liquid chromatography quadrupole time-of-flight mass spectrometry (LC-ESI-Q-TOF). The major phytoconstituents detected in the leaves extract include m-coumaric acid, Robinetinidol-4alpha-ol, C16 Sphinganine, quinic acid, apocynin A, nonic acid, quercitin 3-(6′-acetylglucoside), robinetinidol-4alpha-ol, and isoorientin 6′-O-caffeate [35].

**Scheme 1:**
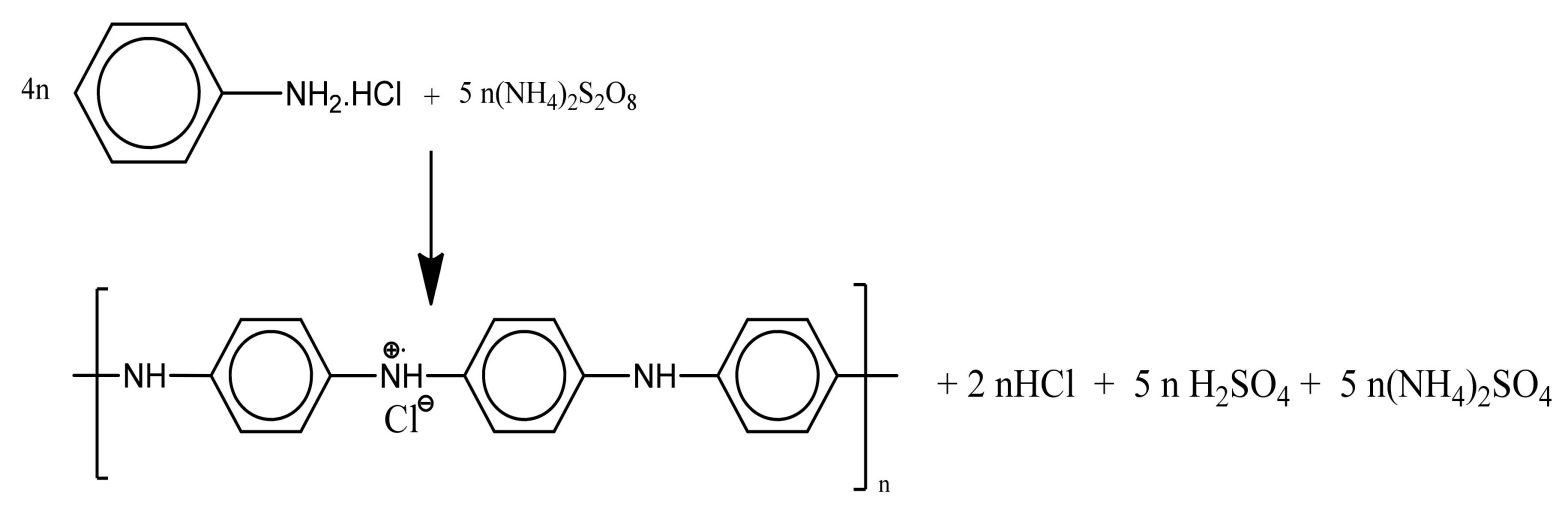
Synthesis of polyaniline.

**Figure 2:**
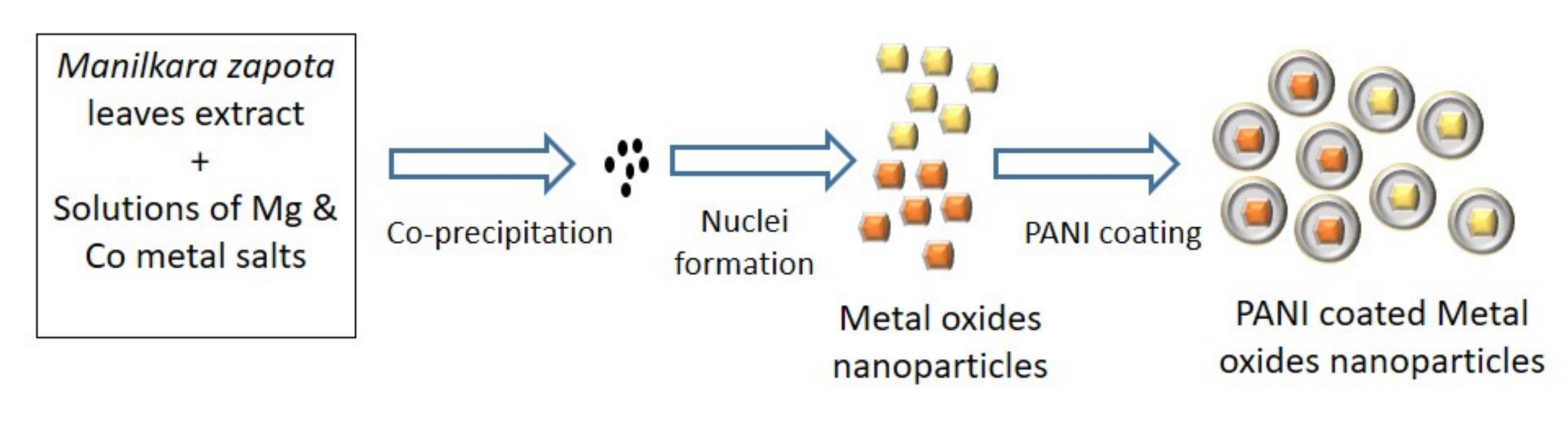
Schematic representation of synthesis of studied nanoparticles.

Though various metallic nanoparticles have been previously studied as antifungal agents, this area is in need of extensive research to improve their efficacy. It is presumed that the nanoparticles disturb the protein structure on the fungal cell surface and trigger the oxidative stress response (ROS). The generation of free radicals by nanoparticles causes oxidation of the lipids present in the fungal cell membrane [36]. Polyaniline is among the class of polymers that exhibit excellent conducting properties [37] and assist the transfer of electrons in polymeric nanoparticles. The surface modification of nanoparticles by polyaniline may further facilitate free radical formation and augment the process of cell death. The chemical reaction of polyaniline formation is given in Scheme 1 [38]. Figure 2 describes the schematic representation of the synthesis of coated particles.

### 3.1. FTIR Analysis

**Figure 3:**
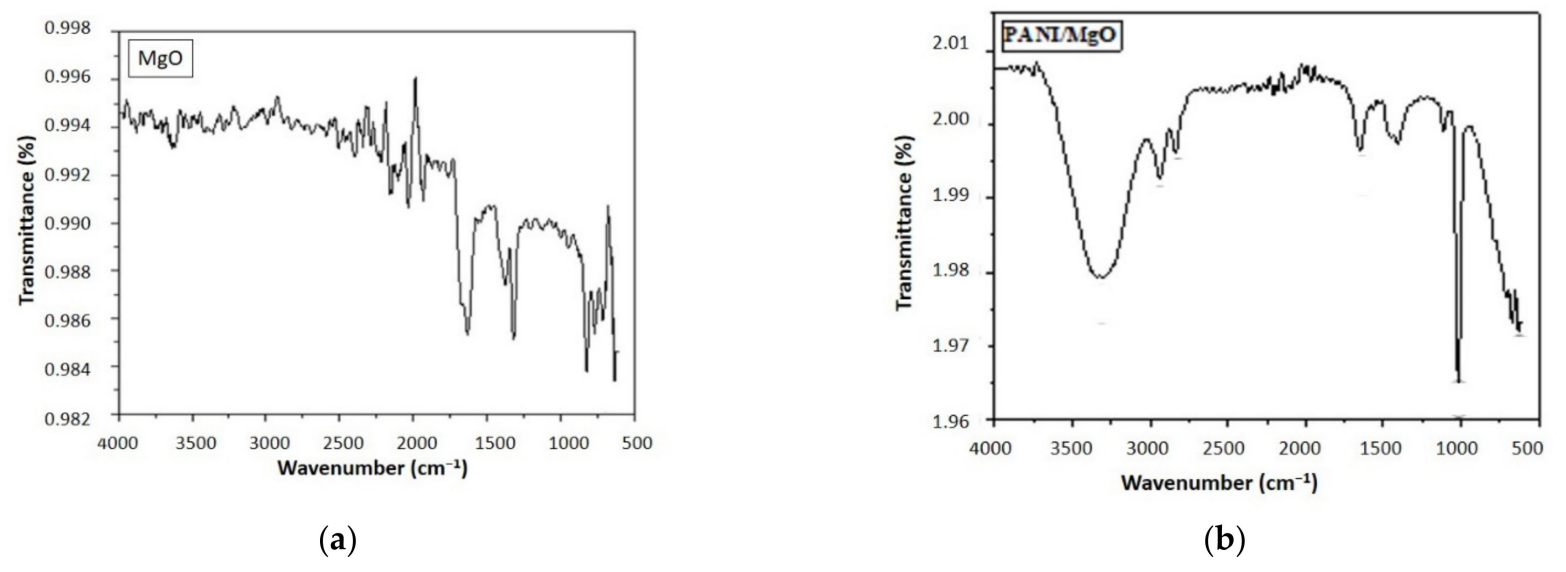
FTIR spectra of (**a**) MgO (**b**) PANI/MgO nanoparticles.

Figure 3a depicts the FTIR spectrum of MgO nanoparticles. The band at 1699 cm^−1^ is assigned to C=O, which possibly appeared due to the surface adsorption and capping of carboxylic acids present in the leaves extract. The strong bands at 1377 and 838 cm^−1^ indicate the Mg-O bond. FTIR spectrum of PANI/MgO (Figure 3b) presents a band at 634 cm^−1^ associated with a C-C bond of an aromatic ring; 3323 cm^−1^ is attributed to an OH band. C-H vibrations (2942 cm^−1^), C=N vibrations (1653 cm^−1^), and a C-N bond (1021 cm^−1^) typical of polyaniline were also observed in the spectrum [39,40].

**Figure 4:**
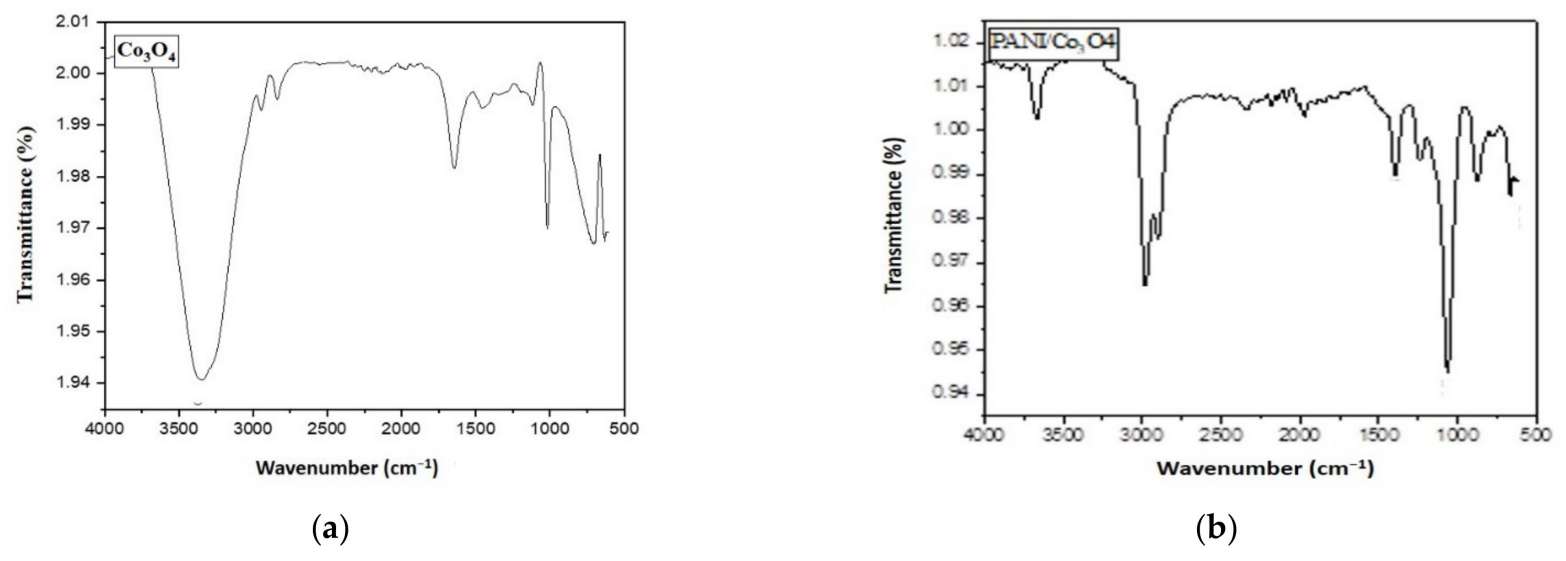
FTIR spectra of (**a**) Co_3_O_4_ and (**b**) PANI/Co_3_O_4_ nanoparticles.

Similarly, the Co_3_O_4_ spectrum (Figure 4a) shows bands at 3343, 2809, 2710, and 1718 cm^−1^, which are attributable to OH, CH_3_, CH_2_, and C=O bonds that resulted due to the capping of carboxylic acids present in the leaves extract. The Co-O bond appeared at 594 cm^−1^.

In the case of PANI/Co_3_O_4_ (Figure 4b), the band at 3650 cm^−1^ describes the presence of an OH bond. The typical characteristic bands of PANI appeared at 3051 cm^−1^ indicative of stretching vibration of aromatic C-H, 2910 and 1370 cm^−1^ representing stretching vibrations of methyl and C-N= groups, respectively. The bands at 1150, 840, and 584 cm^−1^ are relative to CH bending vibration, out of plane bending vibration of aromatic CH and Co-O bonds, respectively [41]. The presence of these functional groups thus confirms the coating of nanoparticles with PANI.

### 3.2. SEM Analysis

**Figure 5:**
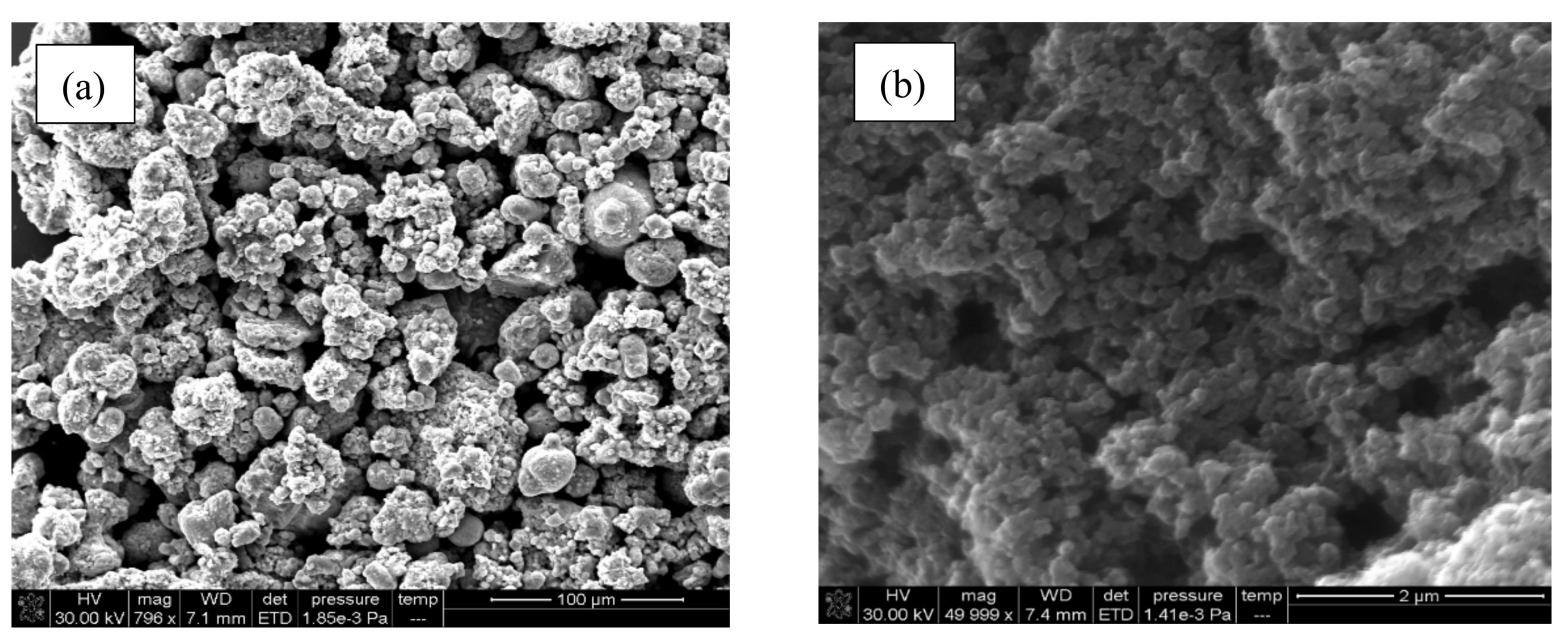
SEM analysis of (**a**) MgO nanoparticles and (**b**) PANI/MgO nanoparticles.

The study of the surface topology of MgO nanoparticles synthesized using *Manilkara zapota* leaves extracts through SEM technique is demonstrated in Figure 5a. The particles appeared spherical and grainy aggregated with homogenous spreading. Figure 5b presents the SEM image of PANI/MgO nanoparticles that shows the uniform spread of spherical particles. The particles appeared to be interlinked with each other forming aggregates.

**Figure 6:**
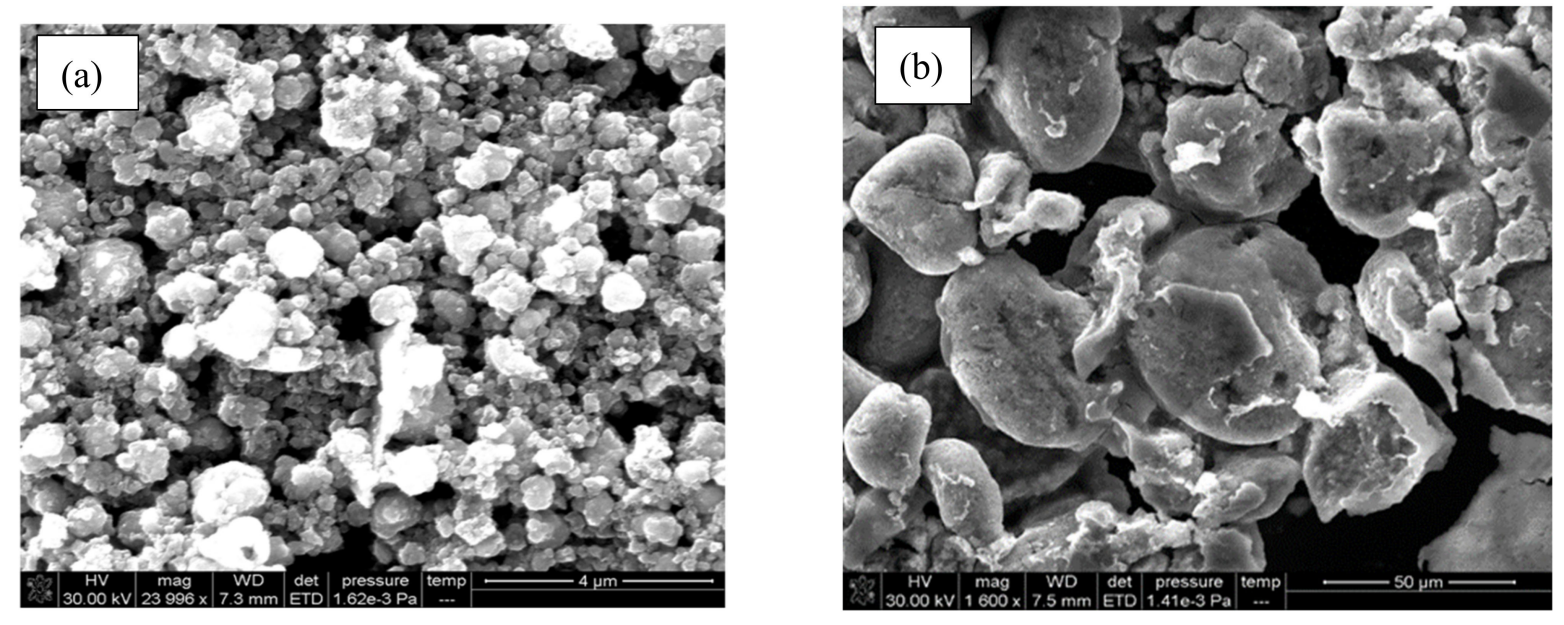
SEM micrograph of (**a**) Co_3_O_4_ nanoparticles and (**b**) PANI/Co_3_O_4_ nanoparticles.

The Co_3_O_4_ nanoparticles appeared as highly aggregated spheres (Figure 6a). While the PANI/Co_3_O_4_ nanoparticles have a relatively reduced aggregation whose possible reason might be the presence of a PANI layer around the particles (Figure 6b) [42].

### 3.3. XRD Analysis

**Figure 7:**
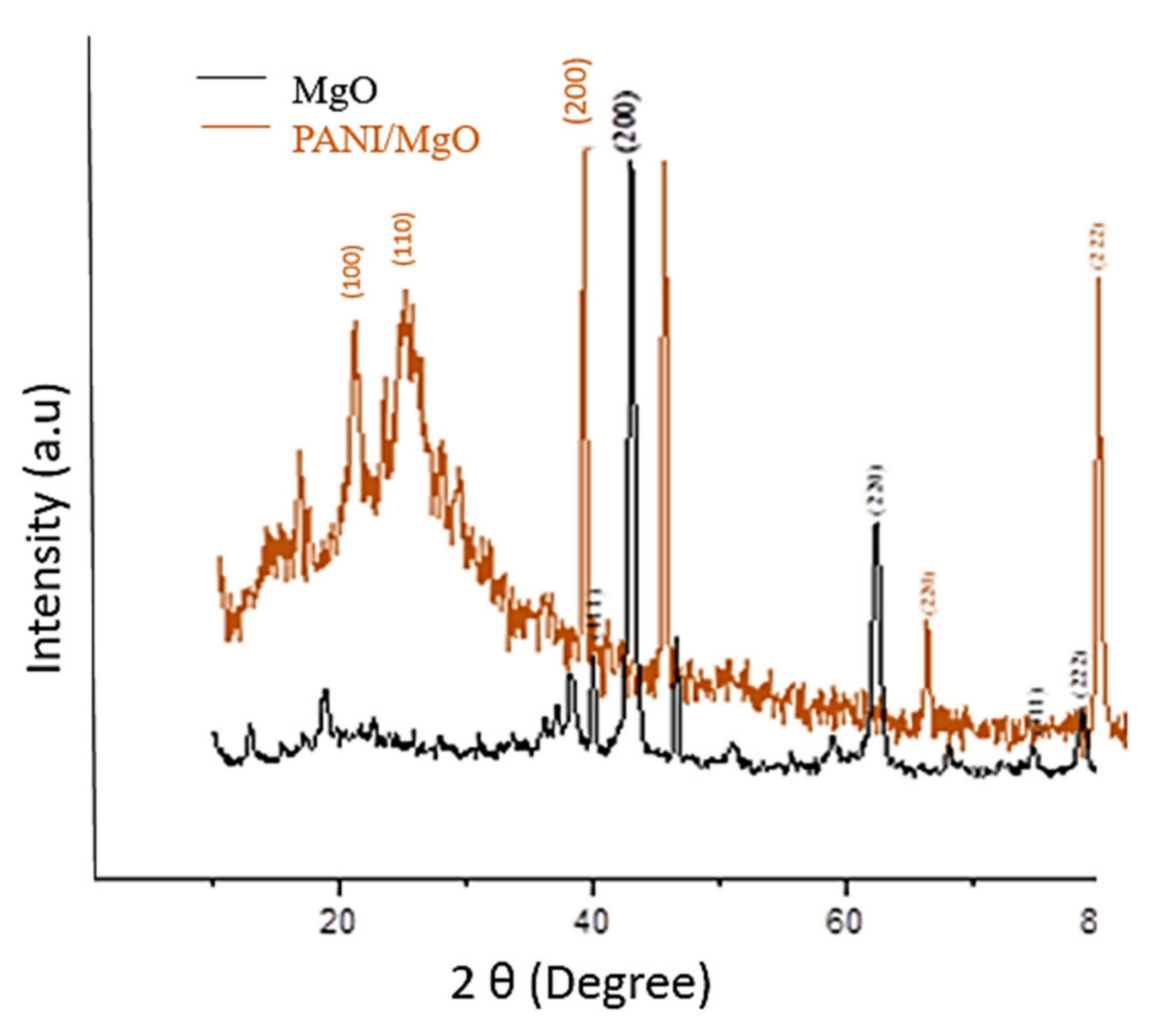
XRD of green synthesized MgO and PANI/MgO nanoparticles.

The XRD spectrum of MgO (Figure 7) presents the peaks at 2 Theta (degree) 37.1°, 43.1°, 62.39°, 74.89° and 78.6° at the planes of (111), (200), (220), (311) and (222), respectively. The XRD spectra show that our desired product was pure magnesium oxide nanoparticles. To determine the crystallite size of modified and unmodified nanoparticles, the Debye Scherrer equation was used:

(1) D=Kλβcosθ

where D is the particle size of material in nm, K is the correction factor and is dependent on crystallite’s gyration radius, λ is the X-ray wavelength, β is the full width at half maximum in radian, and θ is the scattering angle. Hence, the size of MgO was calculated to be 25 nm.

The XRD spectrum of coated MgO with polyaniline is shown in Figure 7. PANI/MgO shows the corresponding peaks of PANI at the plane (100) and (110) and some additional peaks of MgO at the planes of (200), (220), and (222), respectively, at 2θ. All the peaks in the XRD pattern are in agreement with the standard MgO (JCPDS 89-7746) [43]. The coated particles showed a particle size of 60.1 nm. The coated particles thus presented a greater particle size than the non-coated particles.

**Figure 8:**
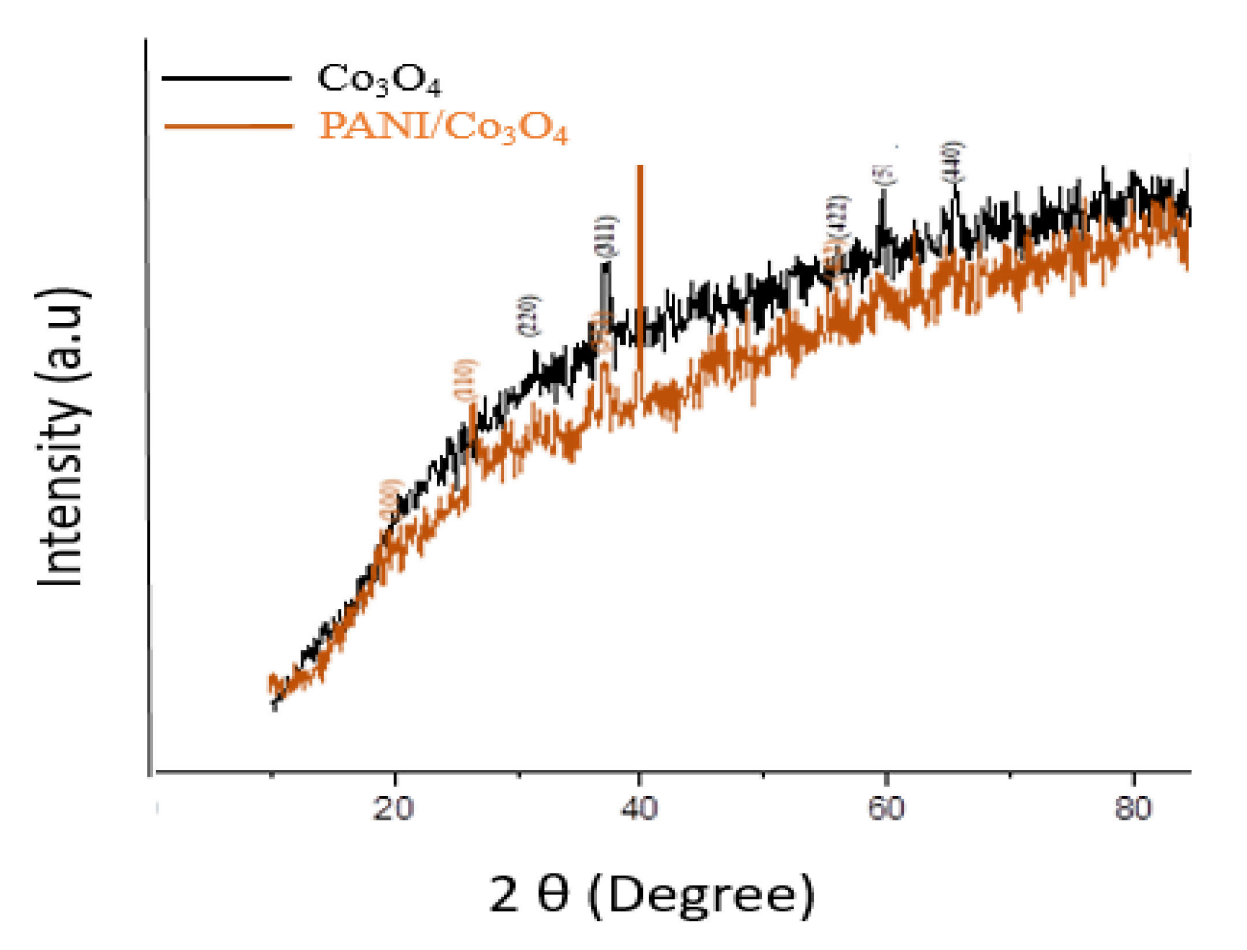
XRD of Co_3_O_4_ and PANI/Co_3_O_4_ nanoparticles.

XRD technique was also utilized to study the arrangement of green synthesized Co_3_O_4_ (Figure 8). Well-defined peaks were not observed in diffraction patterns, which suggests that the obtained nanoparticles do not have a clear crystalline structure [44]. The XRD peaks with diffraction angle of 2θ are 31.3°, 36.93°, 55.7°, 59.3°, 65.3° are related to the planes of (220), (311), (422), (511), (440) respectively (JCPDS: 42-1467) [45]. The average particle size of cobalt oxide nanoparticles was found to be 20.23 nm.

In the case of PANI coated Co_3_O_4_ nanoparticles, the peaks at 2θ are shown at the plane of (100) and (110), which suggest the contribution peaks of PANI. The peaks at the angle of 36.9° and 55.7° at the plane of (311) and (422) are the corresponding peaks of cobalt oxide nanoparticles (Figure 8). The PANI-coated Co_3_O_4_ particles also presented an increased particle size compared to non-coated and were found to be 34 nm.

### 3.4. Zone of Inhibition Test

The ubiquitous existence of pathogenic fungi has been the cause of inextricable havoc on the food industry. The fungi, being capable of genetic modification, often develops resistance to combat fungicides and propagates their species. This has led to nanotechnology paving the way towards the exploration of novel fungicides based on metallic nanoparticles. Metallic nanoparticles are believed to cause membrane disruption of pathogens as a mechanism of cell death [46]. In this study, MgO and ZnO nanoparticles were compared with their respective polymeric nanoparticles. Hence, we expected that the coating of nanoparticles with PANI would further facilitate cell death and enhance the antifungal activity [47,48]. The percentage decrease in fungal pathogens growth was calculated by using the following formula:

(2) $$\mathrm{Percentage}\;\mathrm{decrease}\;\mathrm{of}\;\mathrm{fungus}\;\mathrm{growth}=\frac{\mathrm{dC}-\mathrm{dT}}{\mathrm{dC}}\;\times \;100$$

where dC is the average diameter of fungal colonies in control (cm), and T is the diameter of fungal pathogens in dual culture plate (cm) [49]. In control, distilled water without nanoparticles was added, and thus no zone formation occurred in the control plate.

### 3.5. Concentration and Time Dependent Inhibition

The effect of variation in concentrations of modified and unmodified nanoparticles on the inhibition of fungal growth was investigated. In the case of culture plates containing the nanoparticles, an increase in the particles’ concentration from 3 mM to 24 mM resulted in the successive formation of large inhibition zones with a maximum inhibition at 24 mM. A further increase in concentration did not cause any appreciable inhibition of fungal growth.

**Figure 9:**
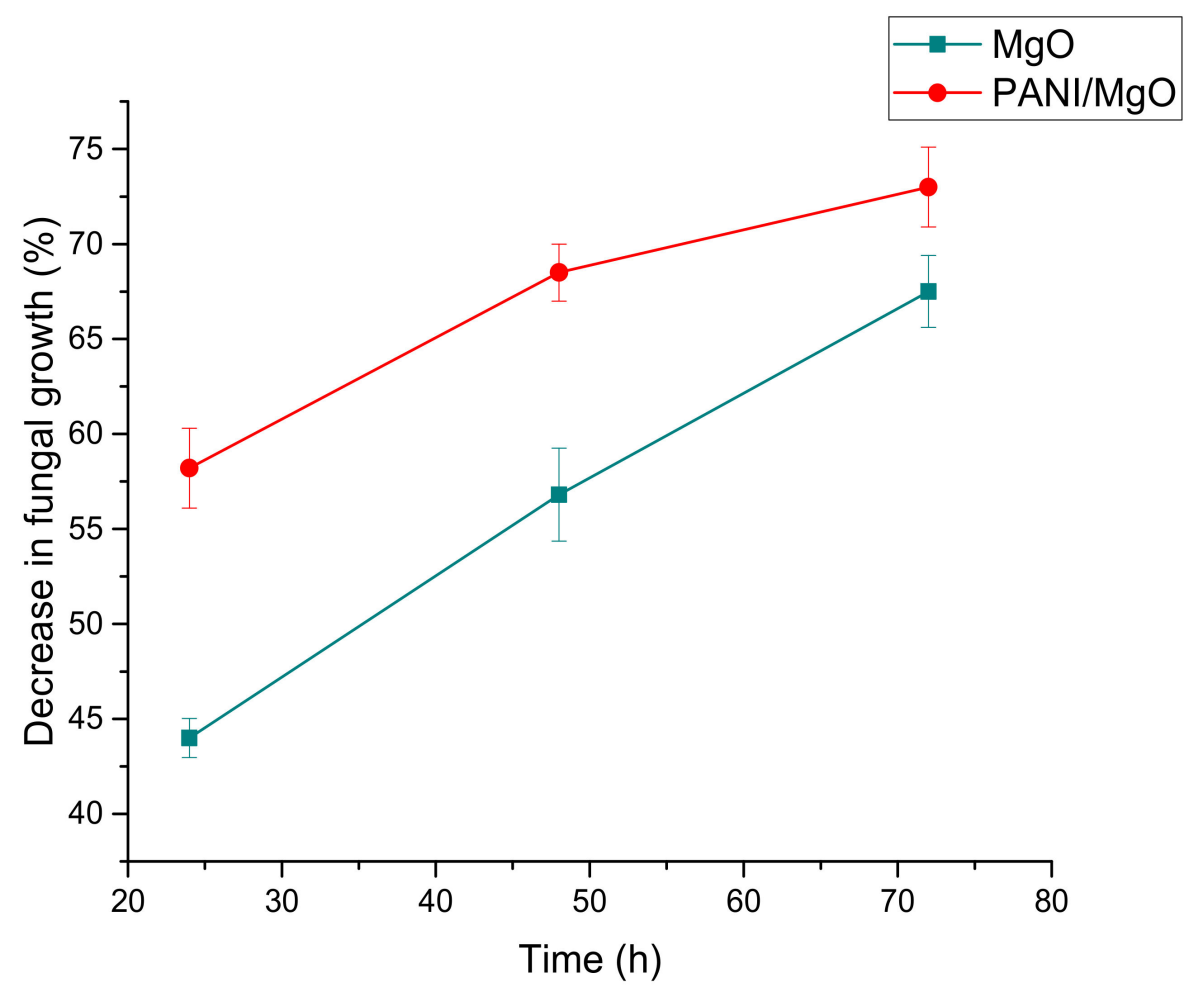
Comparison of the rates of decrease in fungal growth (%) by MgO and PANI/MgO nanoparticles at their 24 mM concentration.

**Figure 10:**
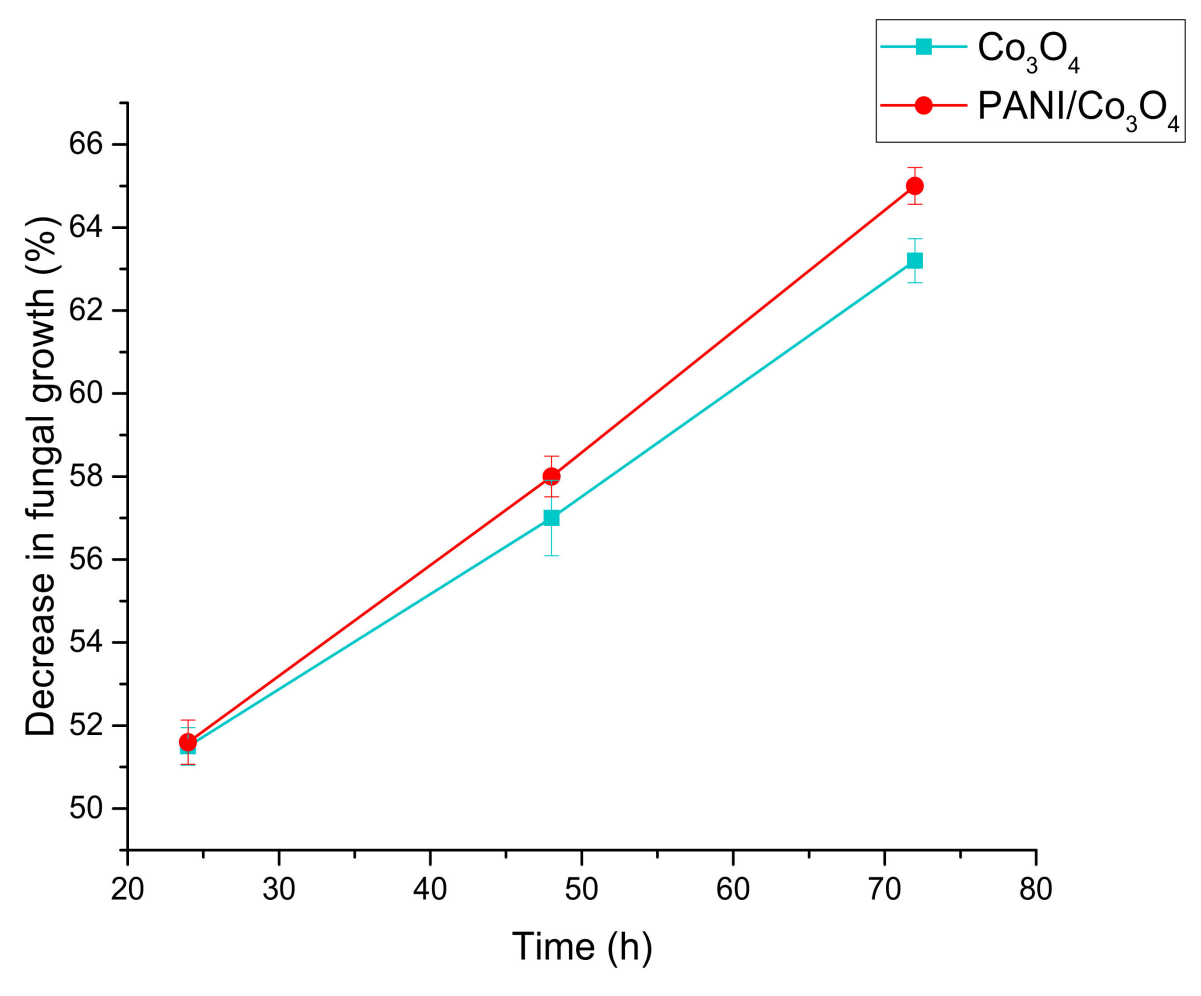
Comparison of the rates of decrease in fungal growth (%) by Co_3_O_4_ and PANI/Co_3_O_4_ nanoparticles at their 24 mM concentration.

Similarly, the exposure time of nanoparticles (modified and unmodified) to fungal strain also predominantly affected its growth. An expansion in the inhibition zone was observed with an increase in time duration (Table 1 and Table 2), which ultimately resulted in a percentage decrease of fungus growth with the passage of time. A maximum zone of inhibition of 2.06 and 2.01 cm was thus obtained at 72 h using 24 mg of PANI/MgO and PANI/Co_3_O_4_ nanoparticles, respectively. All the nanoparticles under investigation showed an inhibition trend. However, coated nanoparticles proved to be stronger than their non-coated counterparts. Based on the inhibition zone, the percentage decrease in fungal growth was calculated for *Aspergillus niger*, and the results of both coated and non-coated nanoparticles were compared (Figure 9 and Figure 10). According to the obtained data, PANI/MgO nanoparticles presented the most effective inhibiting properties and appeared to be powerful antifungal agents in comparison to others under study.

The antifungal ability of the coated nanoparticles was also compared with a standard fungicide, voriconazole. It is a synthetic second-generation triazole that is capable of halting fungal growth effectively and is a well-known fungicide [50]. The zone of inhibition of 4.5 cm was obtained against *Aspergillus niger* after 48 h, which further increased to 4.8 cm at 72 h. The investigated nanoparticles, however, presented promising results, yet further research is needed to improve their antifungal properties.

### 3.6. Performance Evaluation

Though nanoparticles have been exceptionally appreciated as antimicrobial agents, only few nanomaterials exist in the literature which were investigated as antifungal agents against *Aspergillus niger*. Most of the studies used silver nanoparticles to control its growth, as they were considered to possess strong antimicrobial properties. The nanoparticles were prepared through a green approach using *Mentha spicata* and were capable of producing a mean zone of inhibition of 0.6 cm using 25 mg of these silver nanoparticles [51]. Similarly, in another study, silver nanoparticles were synthesized using *Ficus racemosa* fruit extract, and 215 mg L^−1^ of nanoparticles were able to produce a zone of inhibition of 0.3 cm [52].

While evaluating the outcome of the current study, it can be clearly observed that the MgO and Co_3_O_4_ nanoparticles resulted in zones of inhibition of 2.06 cm and 1.92 cm, respectively, which were higher than the literature values for silver nanoparticles. Furthermore, their coated counterparts produced a mean zone of inhibition of 2.16 and 2.01 cm, respectively. This suggests that they were superior in performance to non-coated MgO and Co_3_O_4_ nanoparticles, and also to silver nanoparticles against *Aspergillus niger*. Table 3 presents a performance comparison of synthesized nanoparticles with the reported ones. From the results, it can be inferred that polyaniline-coated metal oxide nanoparticles exhibit good potential as antifungal agents relative to non-coated metal oxide nanoparticles.

## 4. Conclusions

Aiming to explore novel antifungal agents against the *Aspergillus niger*, nanoparticles of magnesium oxide and cobalt oxide were synthesized using an eco-friendly approach and further modified by coating with PANI. The use of *Manilkara Zapota* leaves extracts provided a cost-effective and environmently friendly pathway to the synthesis of MgO and Co_3_O_4_ nanoparticles compared to chemical routes. Taking into account these types of materials, polymeric nanoparticles proved to have greater potential as antifungal agents against the investigated fungal strain. Furthermore, the concentration and exposure time of these nanoparticles also played an effective role in enhancing their antifungal ability. It can therefore be concluded that the nanoparticles investigated in this study can offer an alternative to current technologies employed for preventing food spoilage caused due to Aspergillus.

## Figures and Tables

**Table 1:** Concentration and time-dependent growth inhibition of *Aspergillus niger* by MgO and PANI/MgO nanoparticles.

		MgO Nanoparticles			PANI/MgO Nanoparticles		
Concentration (mM)	Replicates	Time (h)			Time (h)		
		24	48	72	24	48	72
		Zone (cm)			Zone (cm)		
3	R1	0.50	1.10	1.50	0.80	1.30	1.84
	R2	0.90	1.30	1.90	1.40	1.60	1.95
	R3	0.90	1.50	2.00	0.80	2.30	2.34
	Mean	0.76 ± 0.28	1.30 ± 0.20	1.80 ± 0.26	1.00 ± 0.34	1.73 ± 0.51	2.04 ± 0.26
6	R1	0.50	1.10	1.90	0.90	1.30	1.90
	R2	0.70	1.30	2.00	0.90	1.90	2.10
	R3	0.90	1.30	2.20	1.30	1.90	2.36
	Mean	0.70 ± 0.20	1.23 ± 0.12	2.03 ± 0.15	1.03 ± 0.23	1.70 ± 0.34	2.12 ± 0.23
12	R1	0.40	1.10	1.80	0.90	1.30	1.80
	R2	0.70	1.32	2.00	1.40	1.60	2.10
	R3	1.00	1.90	2.10	1.60	1.90	2.10
	Mean	0.70 ± 0.3	1.44 ± 0.41	1.96 ± 0.15	1.30 ± 0.36	1.60 ± 0.30	2.00 ± 0.17
24	R1	0.40	1.10	1.60	1.00	1.80	2.10
	R2	0.90	1.10	2.20	1.30	1.70	2.30
	R3	1.40	1.60	2.40	1.90	1.30	2.10
	Mean	0.90 ± 0.50	1.26 ± 0.29	2.06 ± 0.41	1.40 ± 0.45	1.60 ± 0.26	2.16 ± 0.12

**Table 2:** Concentration and time-dependent growth inhibition of *Aspergillus niger* by Co_3_O_4_ and PANI/Co_3_O_4_ nanoparticles.

		Co_3_O_4_ Nanoparticles			PANI/Co_3_O_4_ Nanoparticles		
Concentration (mM)	Replicates	Time (h)			Time (h)		
		24	48	72	24	48	72
		Zone (cm)			Zone (cm)		
3	R1	0.80	1.10	1.85	0.90	1.10	1.90
	R2	1.00	1.55	1.80	0.72	1.30	2.24
	R3	0.90	1.60	1.98	0.60	2.00	2.05
	Mean	0.90 ± 0.10	1.41 ± 0.27	1.87 ± 0.09	0.74 ± 0.15	1.46 ± 0.47	2.06 ± 0.17
6	R1	0.80	0.95	2.10	0.80	0.95	2.10
	R2	0.90	1.30	1.94	0.70	1.46	1.95
	R3	1.20	1.60	1.92	0.60	2.10	2.10
	Mean	0.96 ± 0.20	1.28 ± 0.32	1.83 ± 0.09	0.70 ± 0.10	1.50 ± 0.57	2.05 ± 0.08
12	R1	0.90	1.10	1.87	0.80	0.90	2.10
	R2	1.00	1.23	1.96	0.63	1.36	2.06
	R3	0.70	1.78	2.10	0.80	1.70	2.27
	Mean	0.86 ± 0.15	1.94 ± 0.36	1.97 ± 0.11	0.74 ± 0.09	1.32 ± 0.40	2.14 ± 0.11
24	R1	0.35	1.13	1.87	0.90	1.10	2.31
	R2	1.00	1.55	1.80	0.70	1.80	1.99
	R3	0.90	1.90	2.10	0.80	1.70	2.30
	Mean	0.75 ± 0.35	1.52 ± 0.38	1.92 ± 0.15	0.80 ± 0.10	1.53 ± 0.37	2.20 ± 0.18

**Table 3:** Comparison of performance of PANI/MgO and PANI/Co_3_O_4_ nanoparticles with reported methods against *Aspergillus niger*.

Nanoparticles	Zone of Inhibition (cm)	Reference
Ag (Green synthesis using *Mentha spicata* leaves extract)	0.6	[51]
Ag (Green synthesis using *Ficus racemosa* fruit extract)	0.3	[52]
PANI/MgO	2.16	Current study
PANI/Co_3_O_4_	2.01	Current study
Voriconazole (Reference fungicide)	4.8	Current study

## Data Availability

The data related to this study is available in the article.
